# Supplementary material for: A method for benchmarking genetic screens reveals a predominant mitochondrial bias
Source: Mol Syst Biol. 2021 May 20;17(5):e10013. doi: 10.15252/msb.202010013 (PMC8138267; doi:10.15252/msb.202010013)
Supplement: Supplementary file 1 — Appendix [file MSB-17-e10013-s002.pdf]

Appendix Figures for:

A method for benchmarking genetic screens reveals a predominant mitochondrial bias

Mahfuzur Rahman[1,*], Maximilian Billmann[1,*,#], Michael Costanzo[2], Michael Aregger[2], Amy H. Y. Tong[2], Katherine Chan[2], Henry N. Ward[3], Kevin R. Brown[2], Brenda J. Andrews[2,4], Charles Boone[2,4], Jason Moffat[2,4], Chad L. Myers[1,3,#]

[1]Department of Computer Science and Engineering, University of Minnesota – Twin Cities, Minneapolis, Minnesota, USA
[2]Donnelly Centre, University of Toronto, Toronto, Ontario, Canada
[3]Bioinformatics and Computational Biology Graduate Program, University of Minnesota – Twin Cities, Minneapolis, Minnesota, USA
[4]Department of Molecular Genetics, University of Toronto, Toronto, Ontario, Canada

[*]Authors contributed equally
[#]Corresponding author
E-mail: chadm@umn.edu, maximilian.billmann@gmail.com

Contents:

Appendix Figure S1: **Overview of FLEX**.

Appendix Figure S2: Mitochondrial complex removal effect on performance of different DepMap CERES score versions across reference standards.

Appendix Figure S3: Protein complex PR contribution.

Appendix Figure S4: Exploration of mitochondrial bias of different DepMap post-processing approaches.

Appendix Figure S5: Exploration of screen size on the performance of FLEX.

Appendix Figure S6: Application of FLEX on chemical genetic CRISPR screens.

Appendix Figure S7: Stability of CORUM protein complexes across three cell lines.

Appendix Figure S8: HAP1 genome-wide CRISPR screen time course data quality.

Appendix Figure S9: Time-resolved CRISPR screens in a single (HAP1) cell line contain a dominant ETC-related functional signature.

Appendix Figure S10: ETC-related signal in RNAi screens.

Appendix Figure S11: DepMap CRISPR screens sorted by complex CERES score.

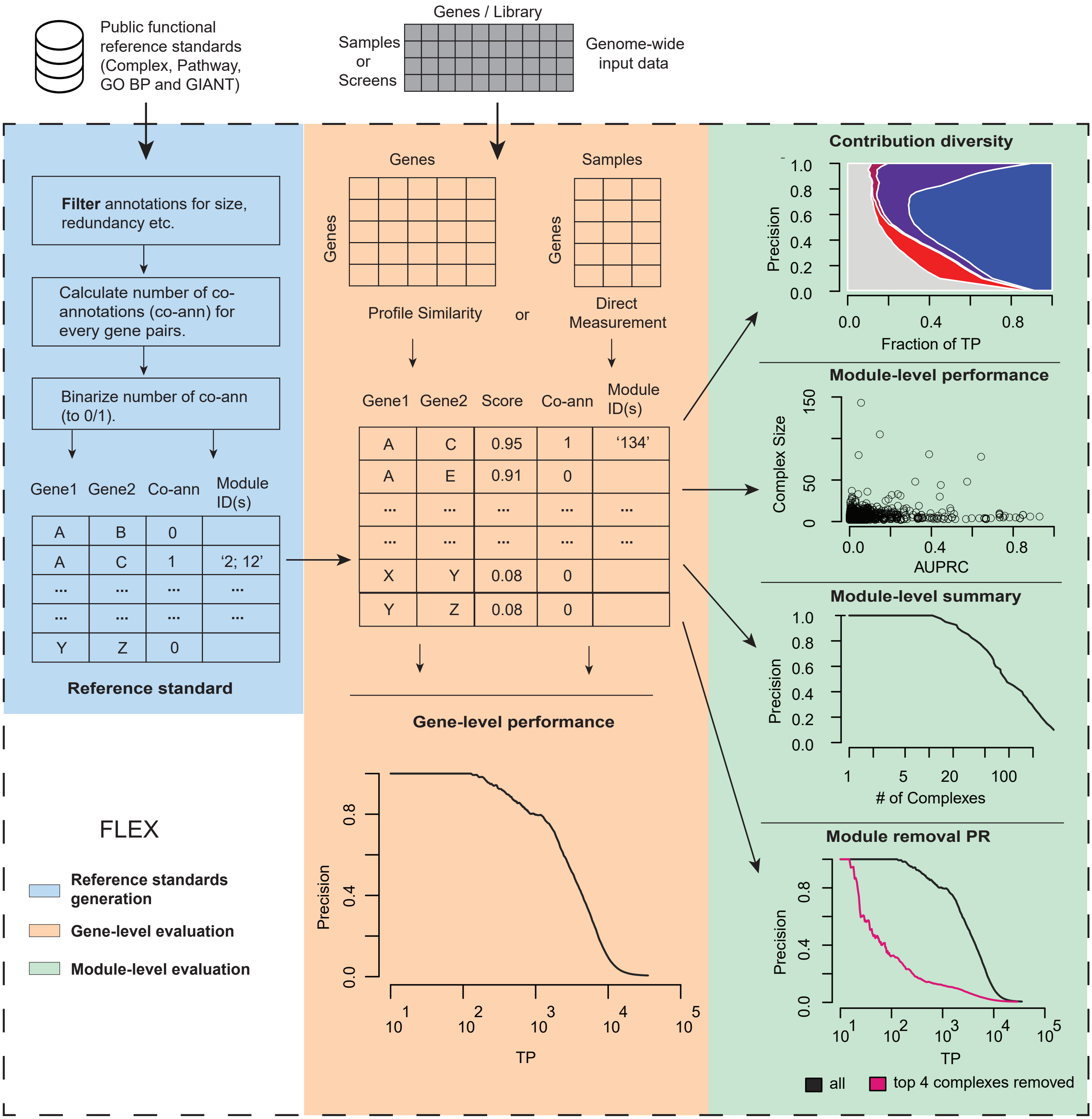

Appendix Figure S1

Appendix Figure S1: **Overview of FLEX**. FLEX investigates and summarizes the functional information in an experiment. **Reference standards generation (light blue)**. FLEX takes as input public functional reference standards and derives a gene-pair binary reference standards for downstream analysis. **Gene-level evaluation (light orange)**. FLEX generates a PR curve summarizing the gene-level (global) performance of the experimental data on CORUM co-memberships. The experimental data can either be dependency scores or genetic interactions. **Module-level evaluation (light green)**. FLEX interprets the module-level (local) functional signal of CORUM complexes. *The contribution diversity* plot summarizes the diversity of protein complexes that contributes to the global PR performance. The top four complexes are highlighted. *The module-level performance* plot visualizes the performance of individual protein complexes. *The module-level summary* plot lists the number of complexes captured at different precision levels. *The module removal PR* plot explains functional bias in global PR performance. The top four complexes are removed from the data and gene-level PR-performance is re-evaluated (pink line).

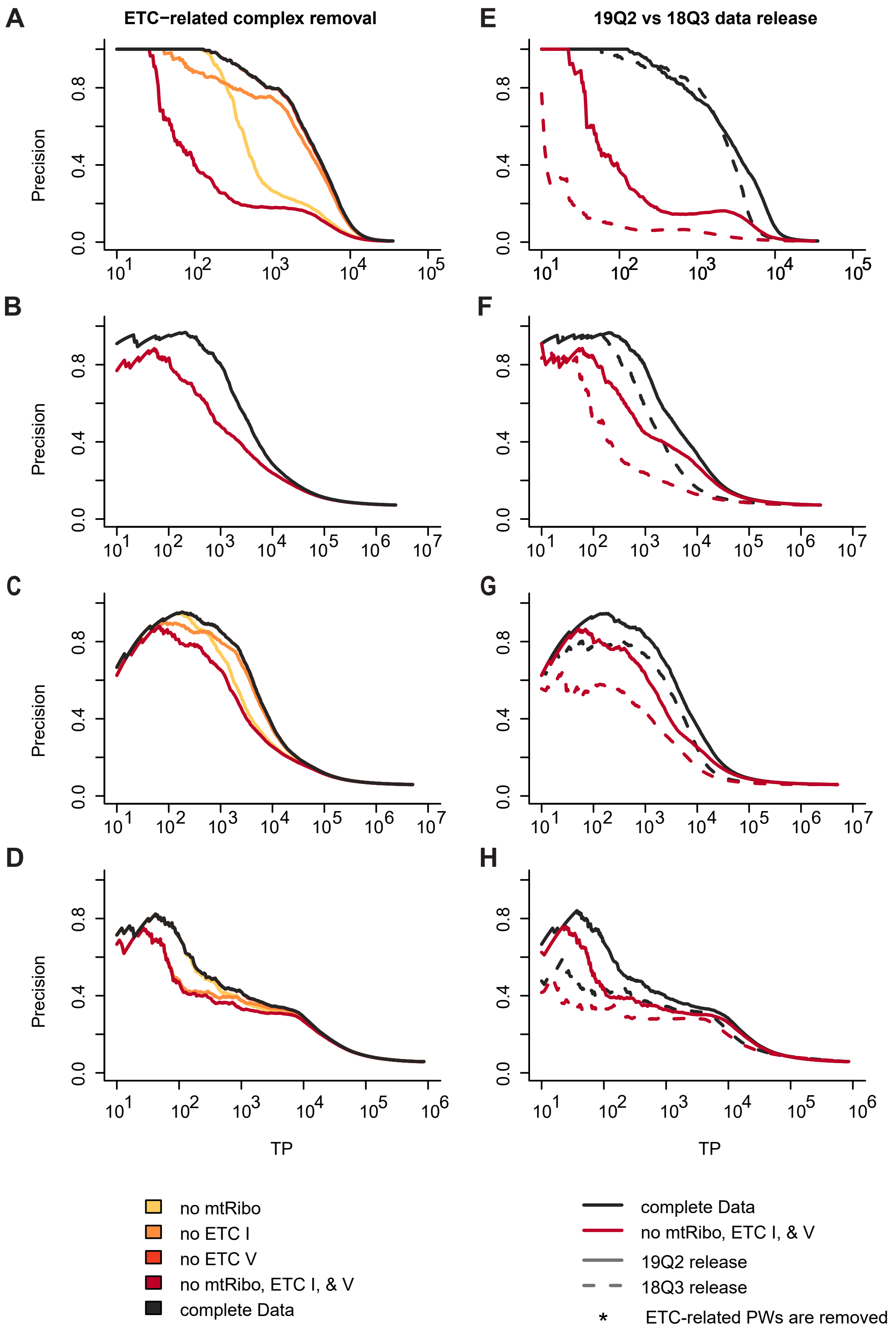

Appendix Figure S2

Appendix Figure S2: Mitochondrial complex removal effect on performance of different DepMap CERES score versions across reference standards. Precision recall curves summarize the performance of DepMap co-essentiality scores to capture functional relationships reported in different standards. **A-D**, Performance decrease of the DepMap 19Q2 release CERES scores after removal of ETC-related complexes. The ETCI (orange), ETCV (light red) and 55S mitochondrial ribosome (yellow) are removed individually and together (dark red) for CORUM, GO BP, and GIANT standards. For Pathway, ETC-related pathways (dark red) are removed (as the Pathway standard does not contain members of the 55S mitochondrial ribosome). Shown is the performance on the standards CORUM 3.0 complexes (A), Pathway (B), Gene Ontology (GO) Biological processes (BP) (C), and the GIANT functional network (D). **E-H**, The performance of DepMap CERES score 18Q3 and 19Q2 releases are compared on the four above-mentioned standards. ETC-related complex removal affects the 18Q3 release performance more strongly than the 19Q2 release performance. The same sets of cell lines from 19Q2 and 18Q3 are used for this comparison.

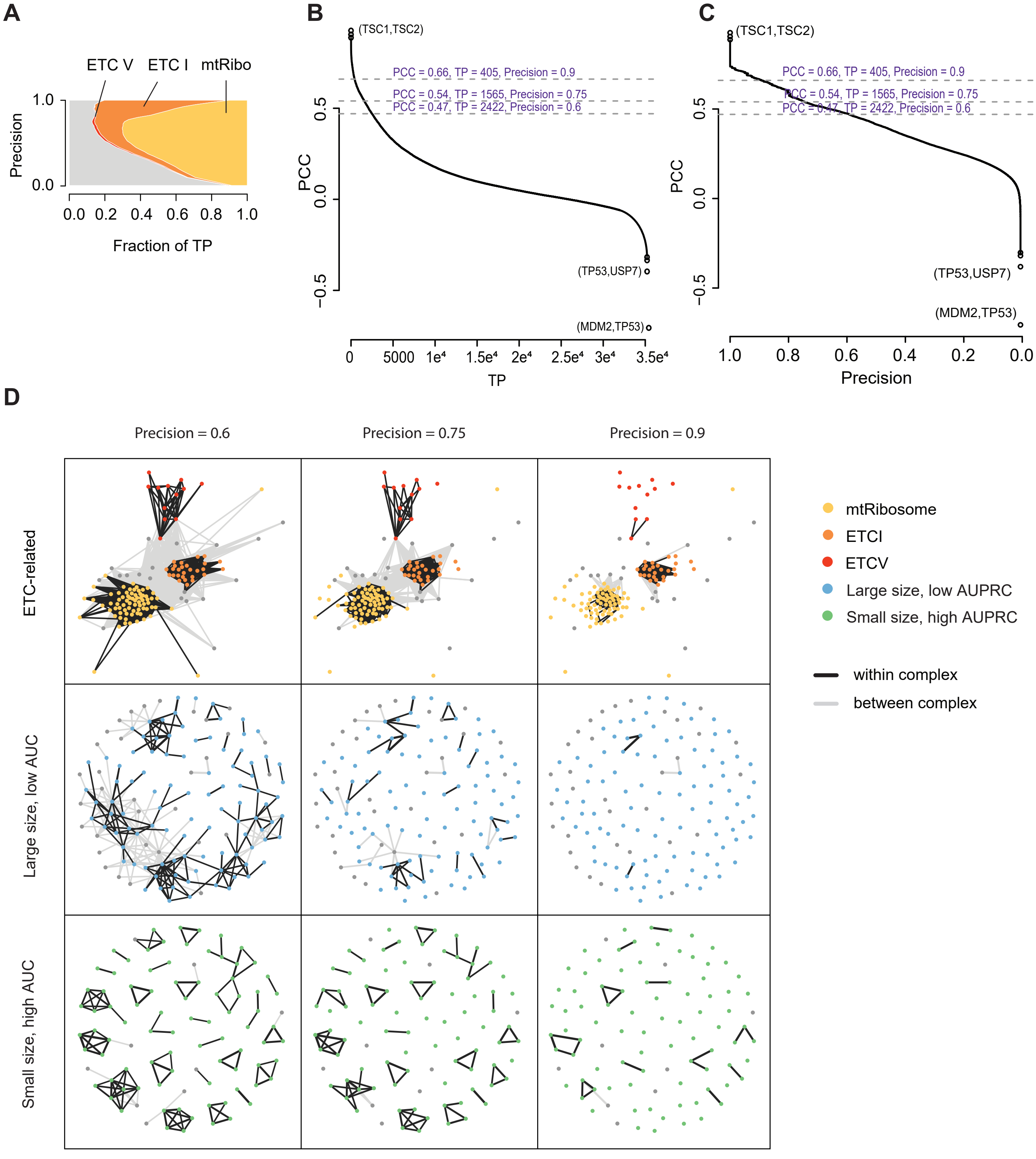

Appendix Figure S3

Appendix Figure S3: Protein complex PR contribution. A, Contribution diversity of CERES score PR performance using the CORUM complex standard with a focus on ETC-related complexes. Shown are the fraction of TP pairs for CORUM complexes at different precision thresholds. **B**, PCC vs TP plot for gene pairs co-annotated to the CORUM complexes. Corresponding precision values for a subset of PCC and TP values are shown. **C**, Similar to **B**, but Precision values are plotted on the x-axis. **D**, Co-essentiality networks using different PCC values as cutoffs (selected at different precision values of 90%, 75% and 60%) and are grouped by ETC-related genes, large size, low AUC genes, and small size, high AUC genes. Gene pairs from within the same complex (TP or co-annotated) are connected through black edges and between complex (FP) gene pairs are connected by light gray edges. Large size, low AUC genes are from complexes with more than 30 members and AUC smaller than 0.4. Small size, high AUC genes are from complexes with less than 30 members and AUC larger than 0.4. ETC-related genes, large size, low AUC genes, and small size, high AUC genes and are color-coded.

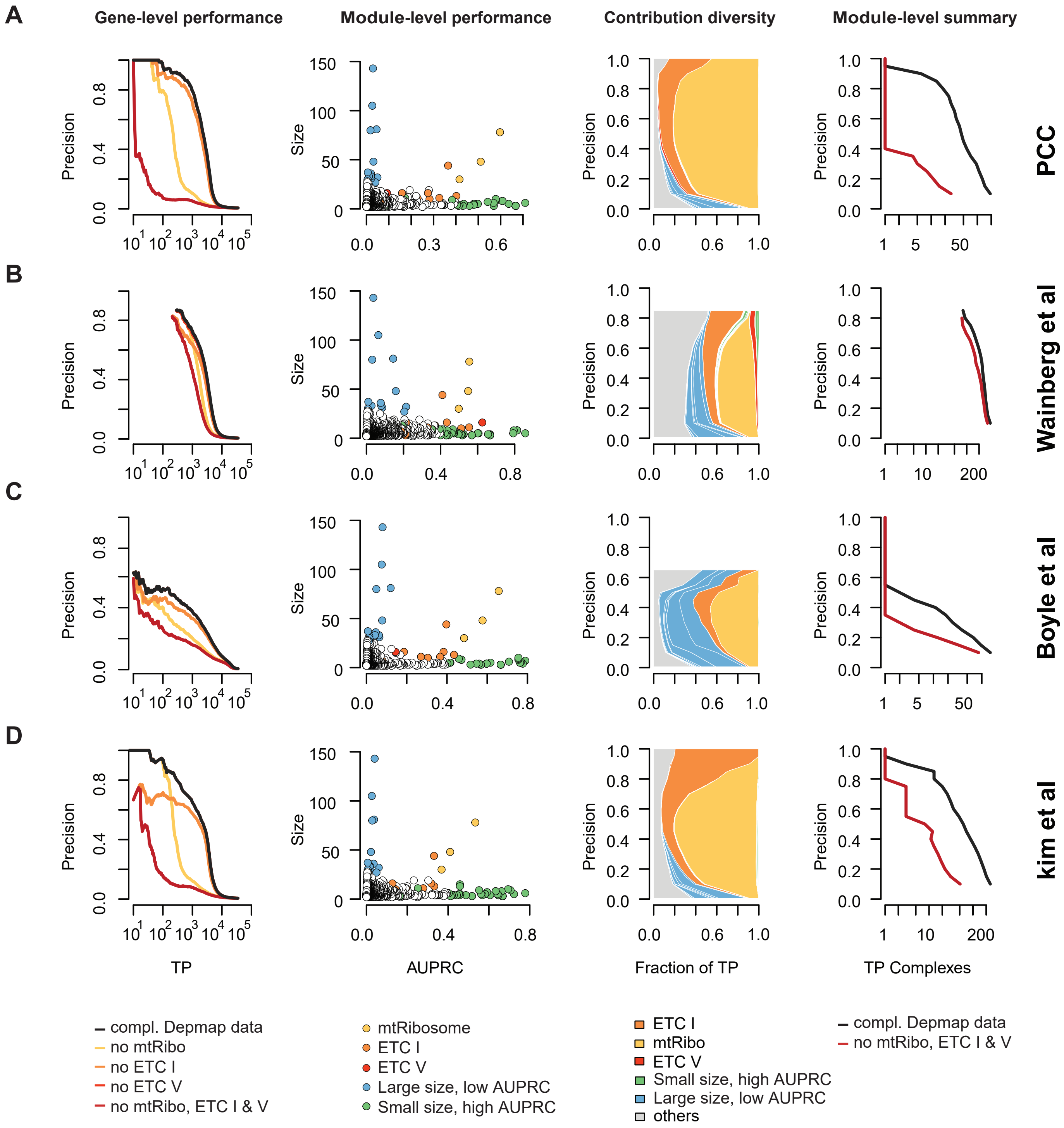

Appendix Figure S4

Appendix Figure S4: Exploration of mitochondrial bias of different DepMap post-processing approaches. Explored are different post-processing methods - for mitochondrial bias - to infer functional relationships from DepMap 18Q3 release CERES score co-essentiality scores; CORUM 3.0 complex relations are used as standard. **A,** Pearson correlation coefficient (PCC) to infer relationships. **B,** Generalized least squares (GLS) based approach by Wainberg *et al*, 2019. This approach bases gene pair similarity scores on FDR corrected p-values (1 - fdr) resulting in a 'late start' of the PR curve (many values at top are the same, 1.0). **D,** CERES score matrix multiplication normalization using olfactory genes to estimate noise in the data by Boyle *et al*, 2018. **D,** PCC-based similarity approach preceded by gene and screen filtering by Kim *et al*, 2019.

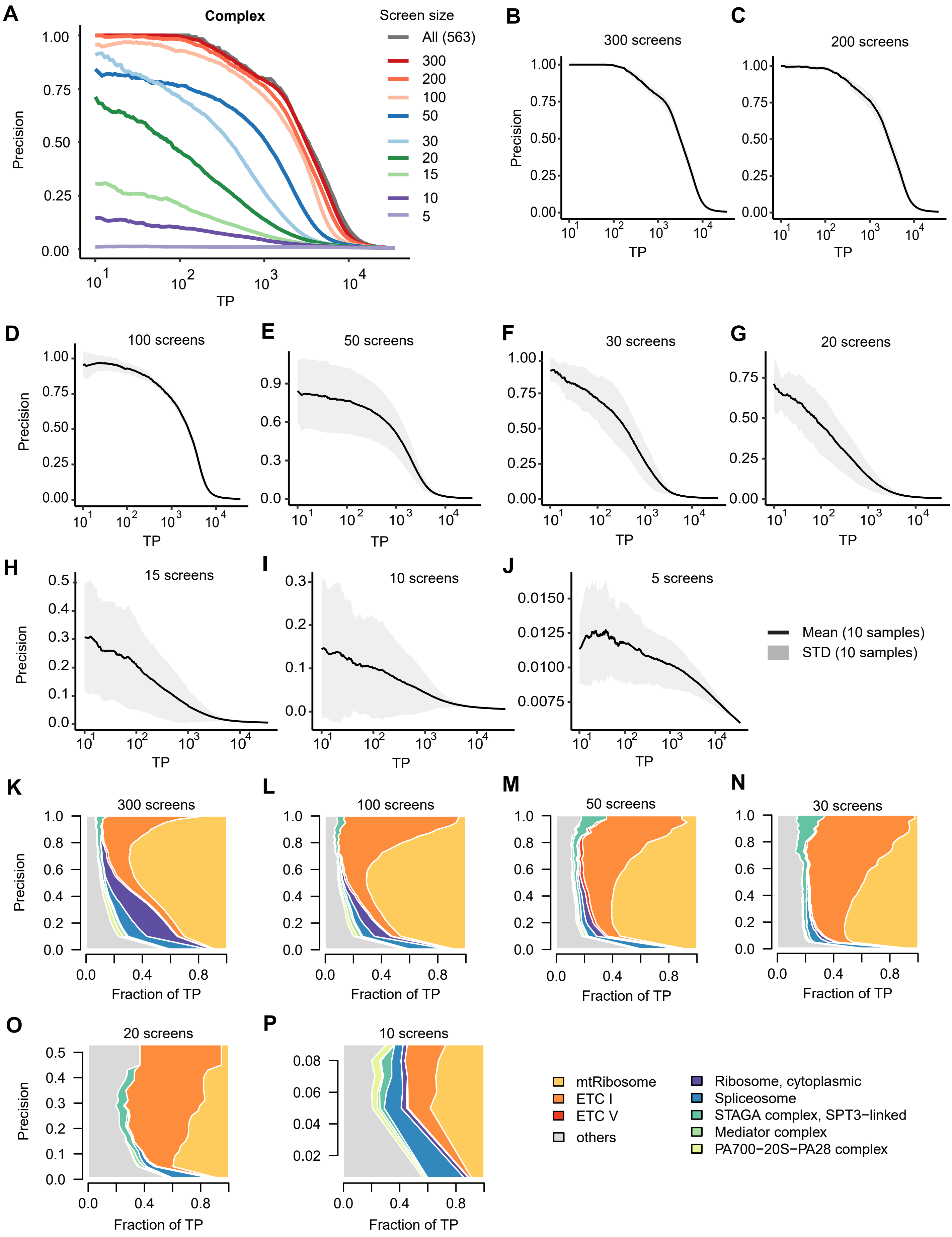

Appendix Figure S5

Appendix Figure S5: Exploration of screen size on the performance of FLEX. Different numbers of screens are subsampled from the Broad DepMap dataset and compared against the full dataset to gauge their ability to infer functional relationships from CORUM complexes. **A**, Gene-level performance comparison to capture co-complex membership on screens of different sizes. **B-J**, Average gene-level performances and their spread for screens of different sizes (300, 200, 100, 50, 30, 20, 15, 10, and 5), each subsampled 10 times. **K-P**, Contribution diversity plots for selected screen sizes; in each case, one of the 10 samples is used.

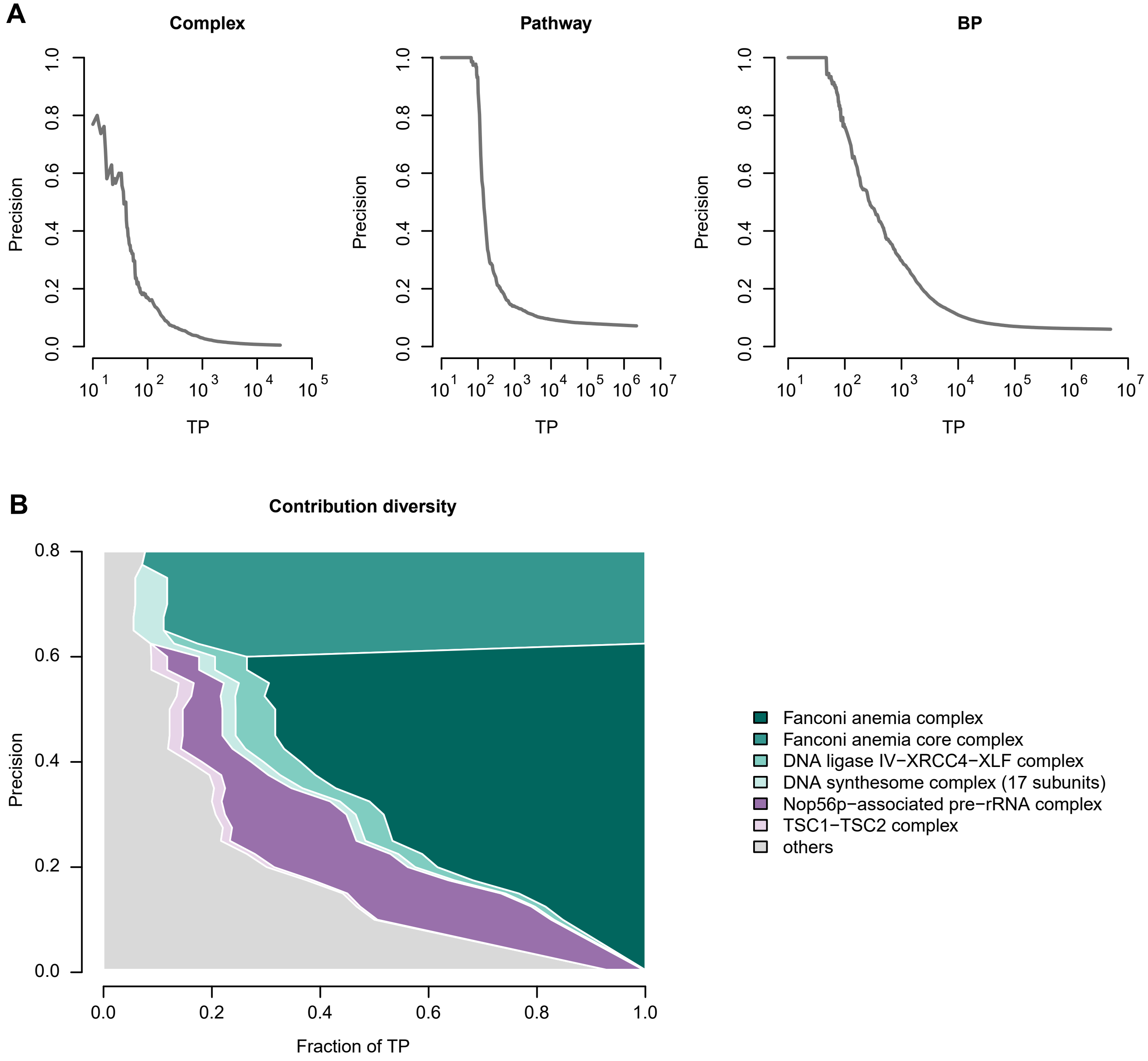

Appendix Figure S6

Appendix Figure S6: Application of FLEX on chemical genetic CRISPR screens. A co-essentiality network is computed from 31 chemical genetics CRISPR screens (Olivieri *et al*, 2020). **A**, Gene-level performance comparison across CORUM, Pathway, and GO-BP standards. **B**, Contribution diversity of CORUM complexes.

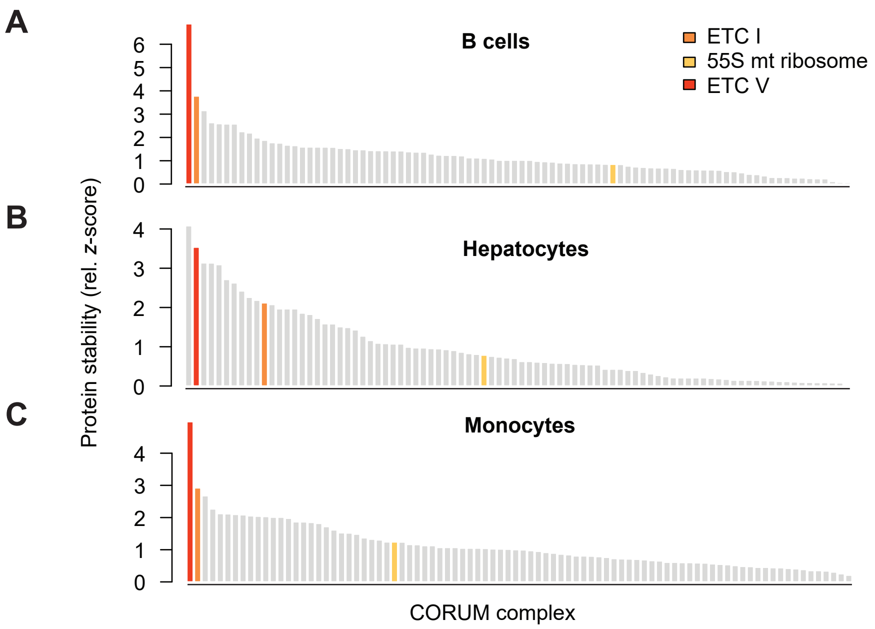

Appendix Figure S7

Appendix Figure S7: Stability of CORUM protein complexes across three cell lines. Shown is the data for B cells (A), hepatocytes (B) and monocytes (C). Protein half-life data was taken from Mathieson *et al*, 2018. The data was summarized on CORUM 3.0 complex level. Half-life data was z-transformed, and the minimum z-score set to 0 to emphasize large z-scores. Complexes for which at least 5 members contributed data across the three cell lines are shown.

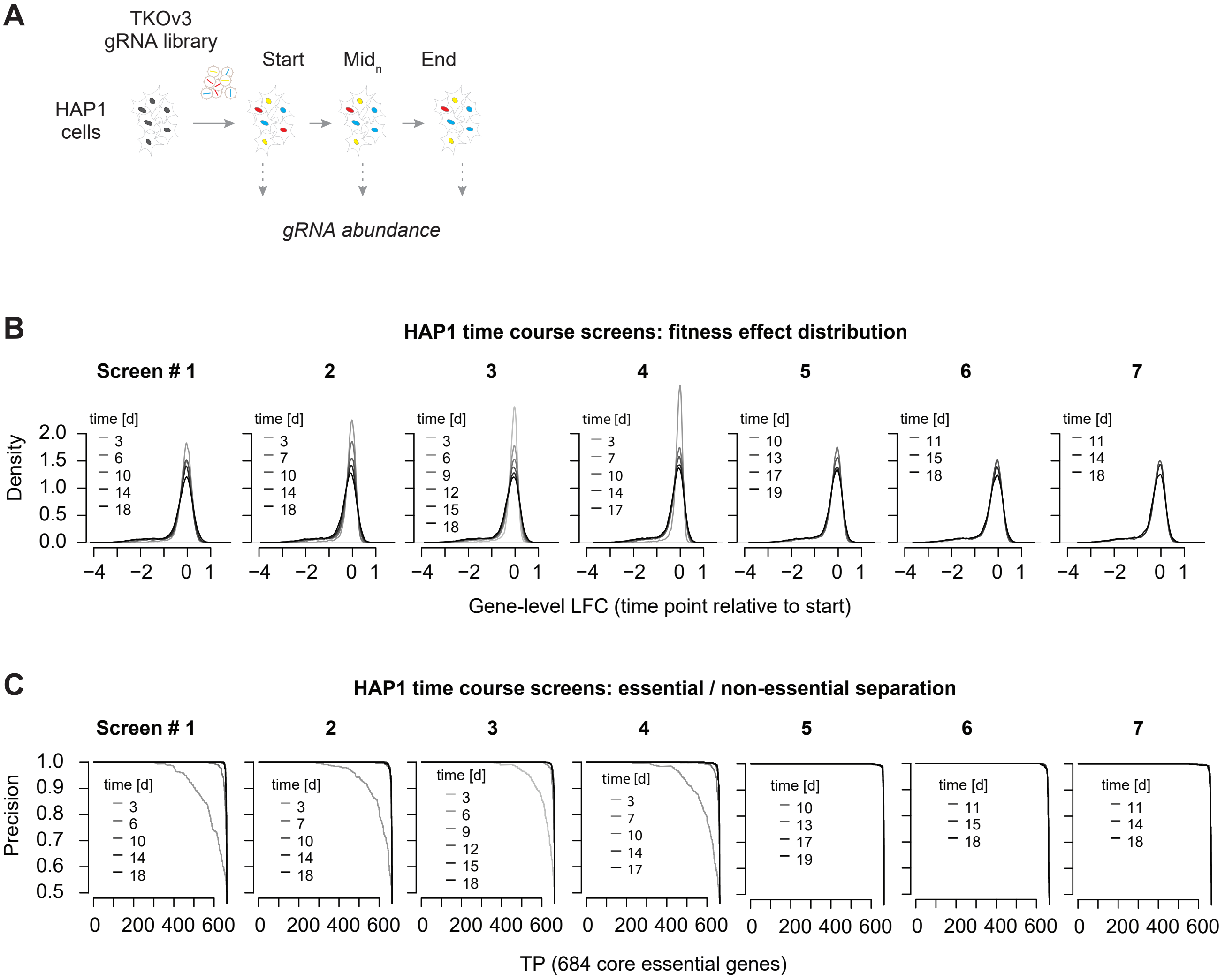

Appendix Figure S8

Appendix Figure S8: HAP1 genome-wide CRISPR screen time course data quality. **A**, Schematic illustration of the experimental workflow for time-resolved genome-wide CRISPR/Cas9 screens in HAP1 cells. **B**, Fitness effect distribution of 17,804 genes targeted with the TKOv3 gRNA library at different time points in the 7 independent screens. The fitness effect is measured by computing a log2 fold-change (LFC) of gRNA abundance at a given time point compared to the starting population (T0; after puromycin selection). **C**, Screen quality control for all time points of the 7 independent screens. This was done by testing the capacity of LFC values to separate 684 core essential and 927 non-essential genes (see Methods).

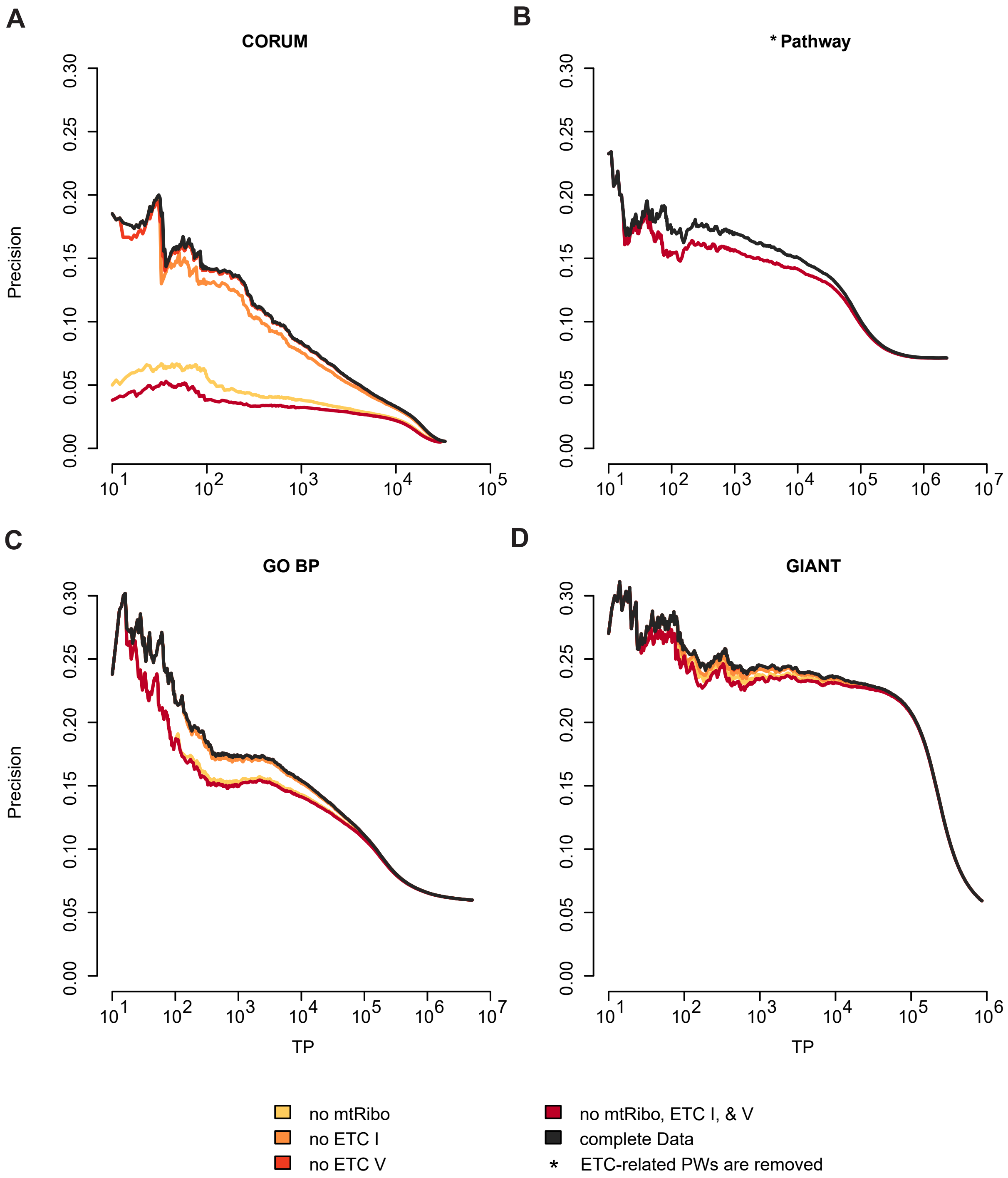

Appendix Figure S9

Appendix Figure S9: Time-resolved CRISPR screens in a single (HAP1) cell line contain a dominant ETC-related functional signature. PR performance of time-course fitness effect profiles in HAP1 cells to capture relationships from CORUM complexes (A), Pathways (B), GO BP (C), and GIANT (D). Pearson correlation coefficients (PCC) are computed between interpolated LFC profiles along the HAP1 time-course. Black line shows complete data, orange, light red and yellow lines show performance after ETC I, V and 55S mitochondrial ribosome removal, respectively. The dark red line shows performance upon removal of all three complexes from the data and standard for CORUM, GO BP, and GIANT. For Pathway, the dark red line shows performance upon removal of ETC-related pathways (the Pathway standard does not contain members of the 55S mitochondrial ribosome).

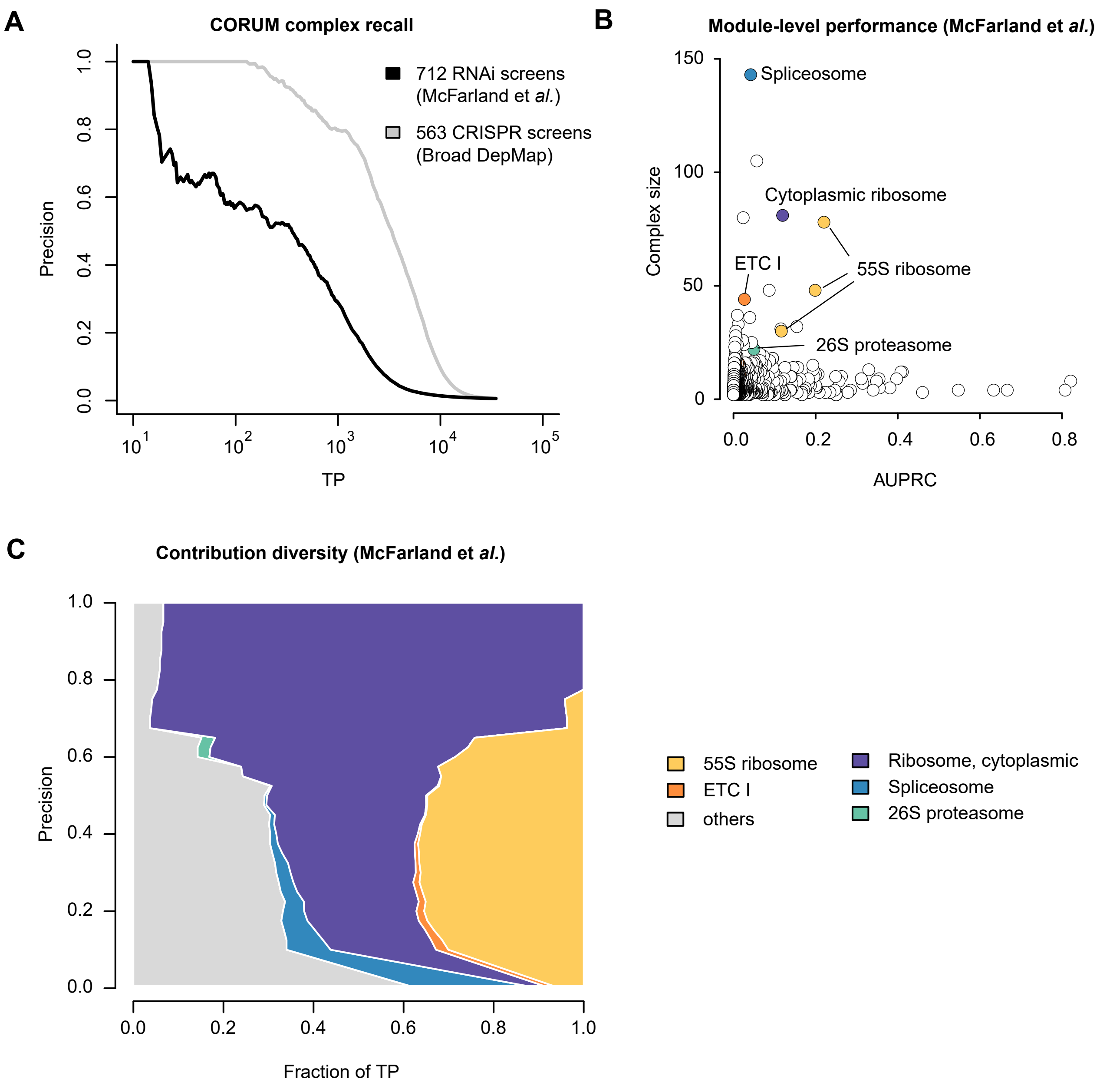

Appendix Figure S10

Appendix Figure S10: ETC-related signal in RNAi screens. Co-essentiality networks are calculated from RNAi (McFarland *et al, 2018*) screens and Broad DepMap (19Q2) CRISPR screens. **A**, Gene-level performance comparison of RNAi and Broad DepMap datasets to capture CORUM co-complex membership. **B,** Module-level performance summary on CORUM complexes. **C**, Contribution diversity of CORUM complexes for RNAi screen data. ETC-related complexes (ETCI, mtRibosome) and selected essential complexes (cytoplasmic ribosome, spliceosome, and 26S proteasome) are highlighted.

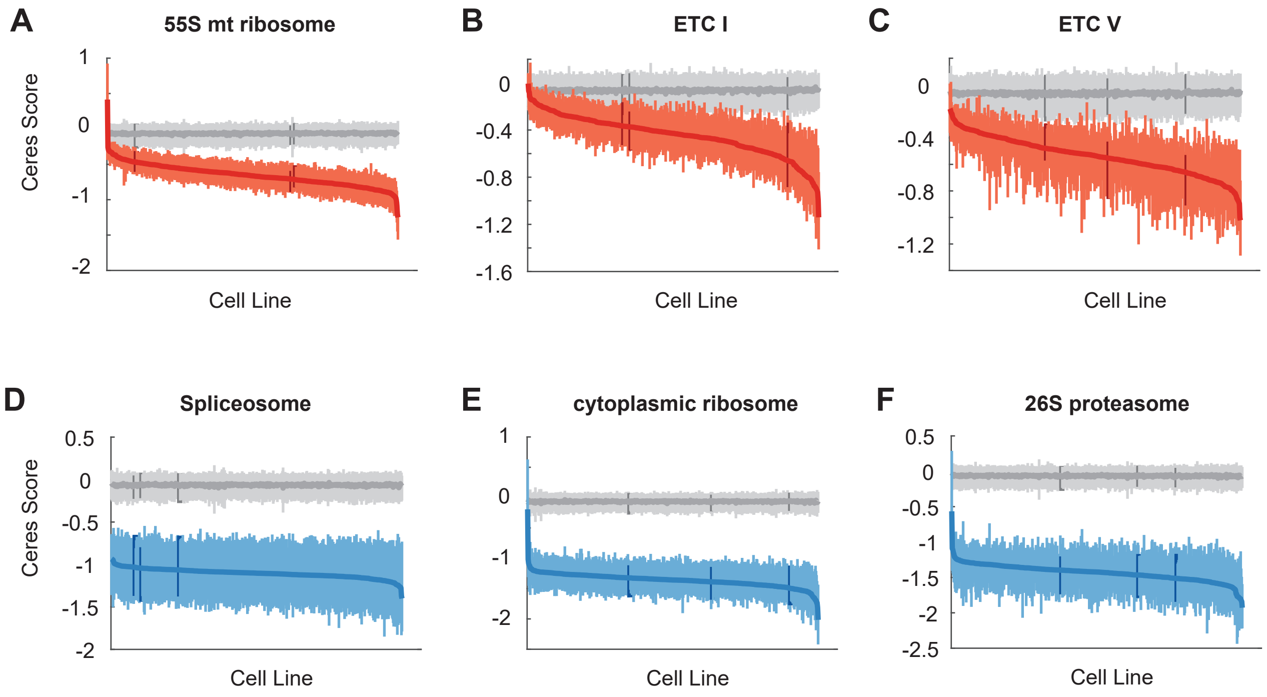

Appendix Figure S11

Appendix Figure S11: DepMap CRISPR screens sorted by complex CERES score. DepMap genome-wide CRISPR screens ranked by the median CERES score across the 55S mitochondrial ribosome (A), the ETC I (B), and V (C) as well as across selected essential complexes including spliceosome (D), cytoplasmic ribosome (E) and 26S proteasome (F). The middle blue/red line indicates the median, the vertical lines the 25% and 75% quantiles of a given screen. Grey lines represent the same metrics for all genes in the genome.

References

Boyle, E.A., Pritchard, J.K. & Greenleaf, W.J. High-resolution mapping of cancer cell networks using co-functional interactions. Mol Syst Biol 14, e8594 (2018).

Kim, E. et al. A network of human functional gene interactions from knockout fitness screens in cancer cells. Life Sci Alliance 2 (2019).

Mathieson, T. et al. Systematic analysis of protein turnover in primary cells. Nat Commun 9, 689 (2018).

Olivieri, M. et al. A genetic map of the response to DNA damage in human cells. Cell 82, 481–496 (2020).

Wainberg, M. et al. A genome-wide almanac of co-essential modules assigns function to uncharacterized genes. bioRxiv (2019).
