# Supplementary material for: A method for benchmarking genetic screens reveals a predominant mitochondrial bias
Source: Mol Syst Biol. 2021 May 20;17(5):e10013. doi: 10.15252/msb.202010013 (PMC8138267; doi:10.15252/msb.202010013)
Supplement: Supplementary file 2 — Expanded View Figures PDF [file MSB-17-e10013-s003.pdf]

Expanded View Figures

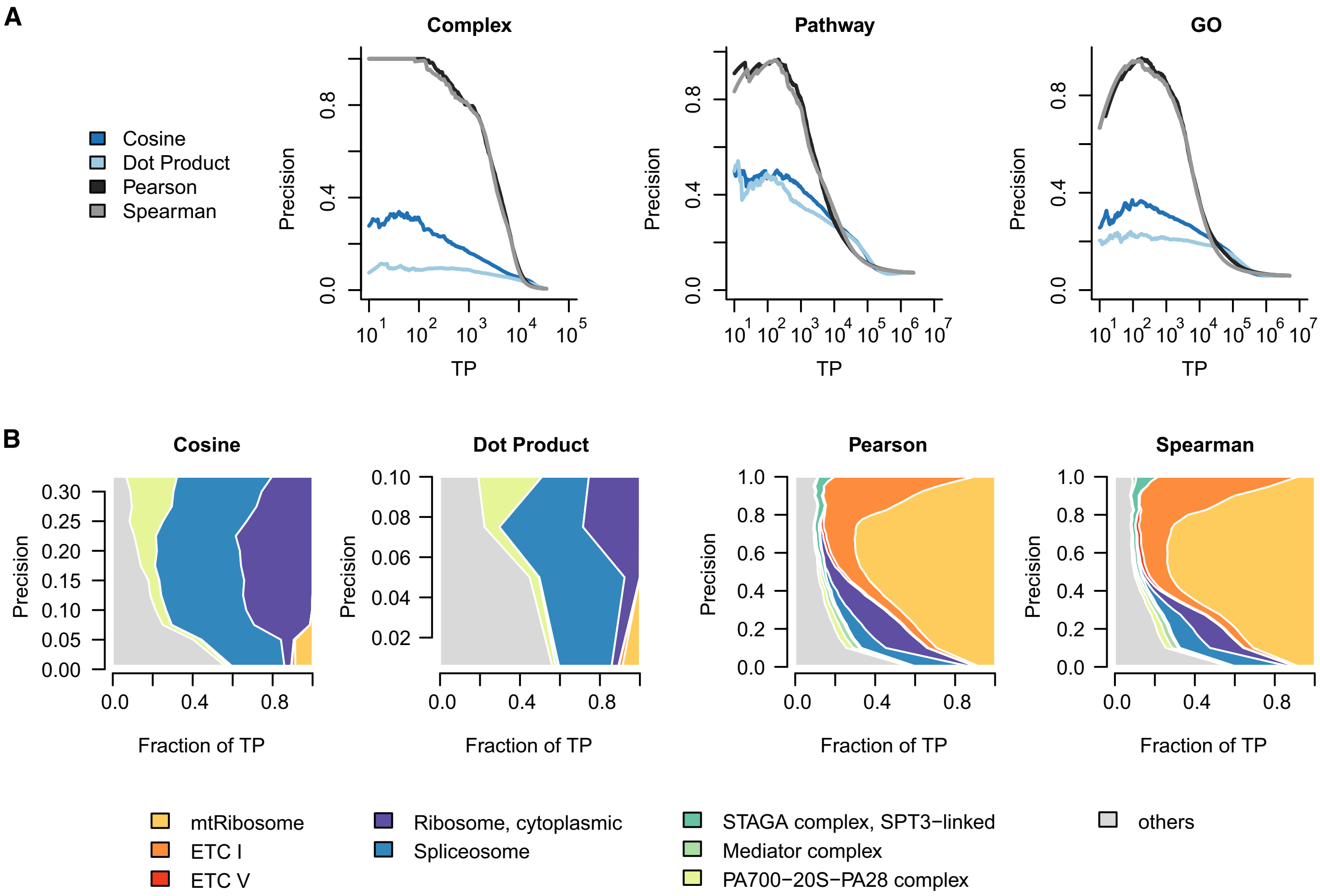

Figure EV1. Effect of similarity measures on functional evaluation.

Co-essentiality networks from the DepMap dataset are calculated using different similarity measures: cosine similarity, dot product similarity, Pearson correlation, and Spearman correlation.

A Gene-level performance comparison of different similarity metrics across CORUM, Pathway, and GO-BP standards.

B Contribution diversity of CORUM complexes for different similarity measures.

© 2021 The Authors

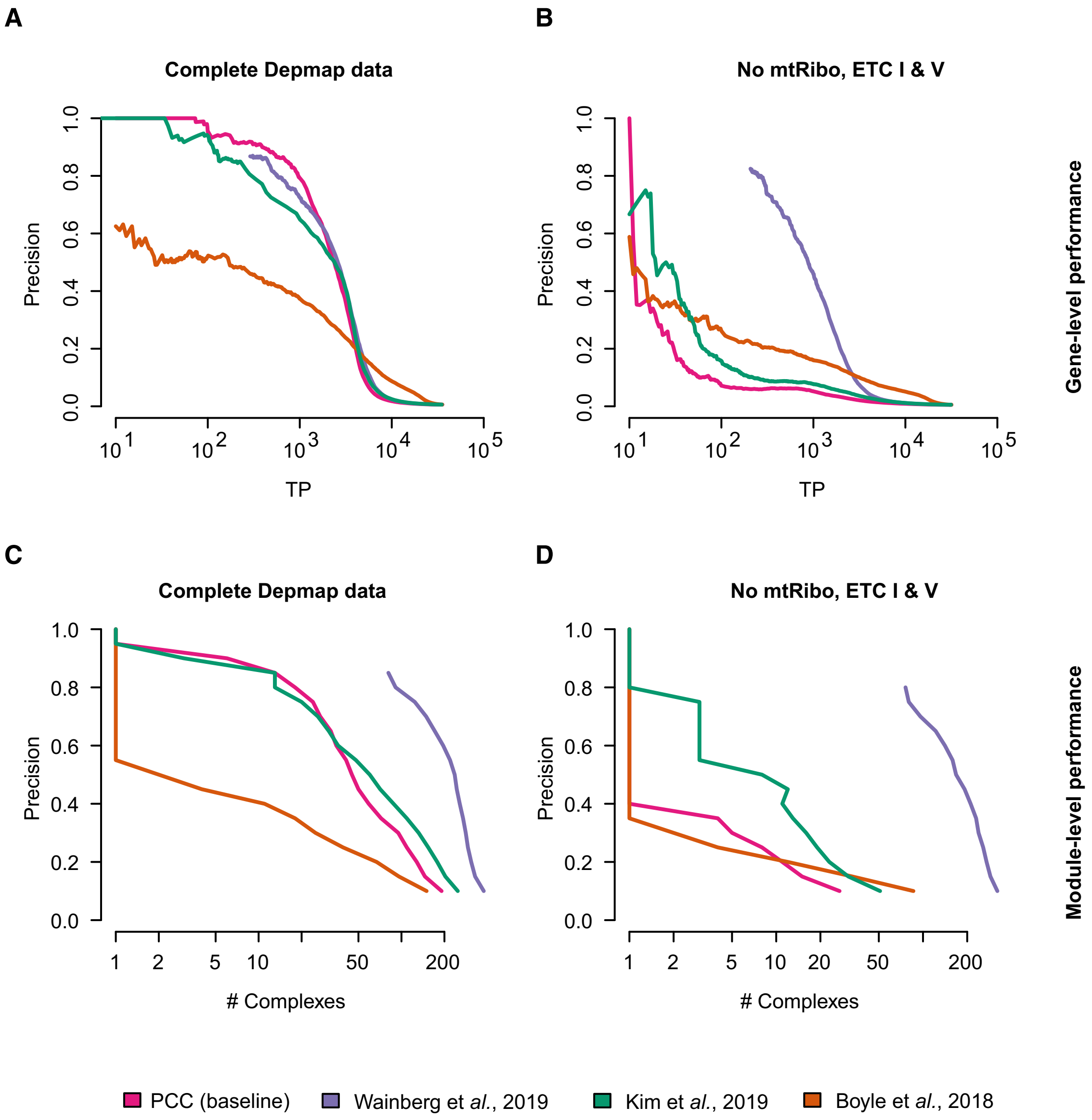

Figure EV2. Direct comparison of alternative DepMap post-processing approaches.

Compared are different alternative methods to infer functional relationships from DepMap 18Q3 release CERES score co-essentiality profiles and CORUM 3.0 complex relations as a standard. The methods are PCC of co-essentiality profiles as baseline data treatment (pink) and approaches published in Wainberg and colleagues (Wainberg *et al*, 2019) (violet), Boyle and colleagues (Boyle *et al*, 2018) (orange) and Kim and colleagues (Kim *et al*, 2019) (green).

A Gene-level performance to capture co-complex membership on the full data.

B Gene-level performance to capture co-complex membership when ETC-related complexes are removed from data and standard.

C Module-level performance to capture number of complexes with TP co-complex membership on the full data.

D Module-level performance to capture number of complexes with TP co-complex membership when ETC-related complexes are removed from data and standard.

 © 2021 The Authors

Figure EV3. Temporal fitness phenotype estimation on HAP1 genome-wide CRISPR screening data.

A Schematic illustration of data processing to estimate dropout rate across time. The contrast of a pair of sliding intermediate time points (Mn, Mm) is used to estimate dropout rates.

B Fitness effects of selected complexes along the time of a screen. Shown is the median gene dropout LFC across a given complex. LFC values for each gRNA are first interpolated from 31 measurements derived from 7 independent screens between 3 and 19 days past puromycin selection (screen start). Next, gRNA-level interpolated LFC values are mean-summarized to the gene-level and finally median-summarized to the complex-level as defined by CORUM 3.0. The range of the sliding intermediate time points is indicated on top.

C Fitness effects of the genes in a selected complex along the time of a screen.

D, E Dropout rate estimation in an early (D) and mid/late (E) window along time. Window size is 3 days (day 4 - day 1 or day 8 - day 5). Gene-level fitness effects (LFC) comparison between sets of time points (left). The differential LFC values (y-axis) depend on LFC values measured at the time point with the strongest phenotypic effect (T18; see Materials and Methods) (middle). This dependency is normalized for, in order to estimate a dropout rate, which is independent of a gene's fitness phenotypic strength (right). Normalization is done by taking the residual from the loess fit. Gray lines show x = y diagonal.

F Adjusted differential fitness effect (negative dropout speed) of genes for each of the selected complexes shown in Fig 2H.

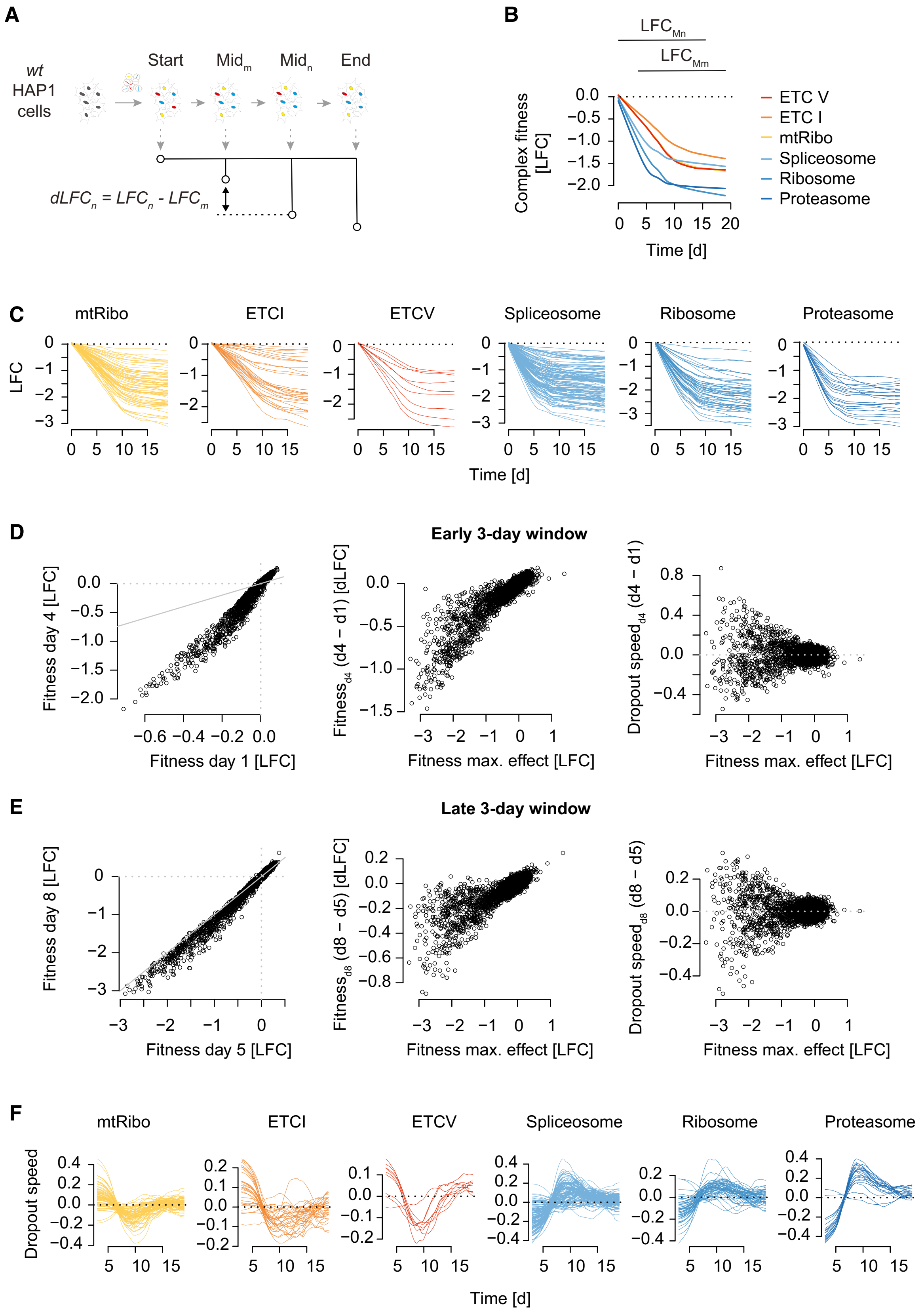

Figure EV3.

 © 2021 The Authors
